# 

**Published:** 1900-06

**Authors:** 


*Vol. XVIII.* *No.* **68.**

LIBRARY
Indexed
S. G. O.

### THE BRISTOL MEDICO-CHIRURGICAL JOURNAL

**A Quarterly Journal of the Medical Sciences for the West of England and South Wales**

*PUBLISHED UNDER THE AUSPICES OF*
*THE BRISTOL MEDICO-CHIRURGICAL SOCIETY.*

LIBRARY

SURGEON GENERAL'S OFFICE

JUL. 9--1900

"Scire est nescire, nisi id me Scire alius sciret."

*Editor:* R. SHINGLETON SMITH, M.D., B.Sc.
WITH WHOM ARE ASSOCIATED
J. MICHELL CLARKE, M.A., M.D.,
J. LACY FIRTH, M.S., M.D., AND JAMES SWAIN, M.S., M.D.
*Assistant-Editor:* P. WATSON WILLIAMS, M.D.
*Editorial Secretary:* JAMES TAYLOR.

BRISTOL: J. W. ARROWSMITH.
LONDON: J. & A. CHURCHILL, 7 GREAT MARLBOROUGH STREET.

*Price One Shilling and Sixpence.*

